# Impact of Giant Cell Arteritis and Its Treatment on the Patient's Quality of Life: A Single-Center Self-Assessment Study

**DOI:** 10.3389/fmed.2021.777310

**Published:** 2021-11-10

**Authors:** Hubert de Boysson, Clivia Barakat, Anael Dumont, Jonathan Boutemy, Nicolas Martin Silva, Gwénola Maigné, Alexandre Nguyen, Amandine Lavergne, Paul Castan, Sophie Gallou, Audrey Sultan, Samuel Deshayes, Achille Aouba

**Affiliations:** ^1^Department of Internal Medicine, Caen University Hospital, Caen, France; ^2^Caen University-Normandie, Caen, France

**Keywords:** giant-cell arteritis, patient report outcome, auto-questionnaire, quality of life, physical disabilities, mental disabilities

## Abstract

Little is known about the impact of giant cell arteritis (GCA) and its treatment on patient-reported physical, mental, and psychic quality of life (QoL). In this monocentric study, a questionnaire was sent to the 100 last patients diagnosed with GCA and followed-up in a single tertiary center. Their physical, mental and psychic status were self-assessed via close-ended questions, the 12-item short form survey (SF-12) and the 15-item geriatric depression scale (GDS). We aimed to identify parameters that were significantly associated with moderate-to-severe disability in both physical and mental domains. Ninety patients were analyzable. Moderate to severe physical disability was found in 41 (46%) patients. In multivariate analysis, walking difficulties (OR, 95% CI 8.42 [2.98–26.82], *p* <0.0001), muscle mass and strength reduction (OR, 95% CI 4.38 [1.37–16.31], *p* = 0.01) and age >80 (OR, 95% CI 4.21 [1.44–13.61], *p* = 0.008) were independent findings associated with moderate to severe physical disability. Moderate to severe mental disability was found in 30 (33%) patients. In multivariate analysis, depressive mood (OR, 95% CI 11.05 [3.78–37.11], *p* < 0.0001), felt adverse events attributable to glucocorticoids (OR, 95% CI 10.54 [1.65–213.1], *p* = 0.01) and use of immune-suppressants (OR, 95% CI 3.50 [1.14–11.87], *p* = 0.03) were independent findings associated with moderate to severe mental disability. There was a statistically significant negative correlation between GDS and the physical and/or mental disability scores (GDS and PCS-12: *r* = −0.33, *p* = 0.0013; GDS and MCS-12: *r* = −0.36, *p* = 0.0005). In conclusion, this study identified via a self-assessment of patients with GCA some medical and modifiable findings that significantly affect their physical and mental quality of life. A better knowledge of these factors may help improve the care of GCA patients.

## Introduction

Giant cell arteritis (GCA) is the most frequent systemic vasculitis, typically affecting patients over 50. The mean age of GCA diagnosis in different studies ranges between 70 and 80 years old (1). The disease burden includes a chronic course and a subsequent prolonged treatment (2, 3), especially because of a high risk of relapse that affects approximately half of patients (4). Glucocorticoids (GCs) remain the cornerstone of treatment, and recent studies have indicated that their management has not significantly changed over the last six decades (3, 5, 6). The GC duration still ranges between 2 and 3 years (7, 8) and is associated with many GC-related side effects. Taken together, the disease and its symptoms, the chronic course and the treatment probably have an impact on the patients' quality of life (QoL), but few studies have been dedicated to this description. Medical consultations during the follow-up of a GCA patient are relatively time-limited and mostly focus on the evaluation of disease activity and treatment tolerance, both being mainly analyzed from a medical point of view.

In this study, we aimed to describe though a self-evaluation methodology, the impact of GCA and its treatments on the patients' QoL, including both physical and mental domains. Using validated scores and scales, we distinguished patients describing a modest impact of the disease and its treatment on their QoL from those with an important impact. From a comparison of these two groups, we sought to identify the factors that most significantly affected their QoL.

## Patients and Methods

### Patients

All patients diagnosed with GCA and followed up in our department are included in a centralized database, and since 2015, data about each patient have been included prospectively.

From our centralized database, we retrieved the 100 last patients consecutively diagnosed with GCA in our department before 31 January 2020. In June 2020, we sent them a paper questionnaire with a stamped addressed envelope to favor returns. GCA diagnosis relied on usual criteria for the disease, including vasculitis demonstration either on the temporal artery by ultrasonography-Doppler or temporal artery biopsy and/or on the aorta and its branches by large-vessel imaging (9, 10). All patients had a regular follow-up in our department, even in the few years following GC discontinuation.

Two months after mailing the questionnaire, patients who did not respond were called on the phone. Missing information in the questionnaire was also retrieved by a systematic phone call to the patient.

The autoquestionnaire was joined to an information note explaining the objectives of the study and specifying that patients could refuse to participate. Patients who returned the questionnaire agreed to participate and gave a written informed consent.

This study was conducted in compliance with good clinical practices and the Declaration of Helsinki principles. At the time of this study, in accordance with French public health law (Art. L 1121-1-1, Art. L 1121-1-2), formal approval from an ethics committee was not required for this type of observational study. Our local ethics committee (Caen CLERS) confirmed the observational non-interventional nature of our work.

### Items Included in the Questionnaire and Studied Parameters

The main objective of the questionnaire was to assess, according to the own point of view of the patients, with the possible contribution if necessary of their family caregiver, how the disease and its treatment have affected their daily life.

The questionnaire included three distinct parts. Part II and III of the questionnaire we sent to the patients is available as a supplementary material.

The first part, not reported in the present article, regards disease manifestations and clinical symptoms assessed by the patients themselves (with the possible help of their caregivers). The second part of the questionnaire regards the GC and their attributable effects. The patient-reported GC tolerance was assessed via questions that focused on eight main areas that we selected as potentially affected by the treatment: metabolic, cardiovascular, muscular, bone, cutaneous and pilar, ophthalmologic, infective, or neurocognitive and psychological complications. Patients were asked to check items in a list of predefined symptoms attributable to the disease or to GC, only if they appeared at GCA onset, during the follow-up or after GC introduction. Symptoms that preexisted before GCA were in theory not checked. In this second part, the GC-related side effects were analyzed according to the disease and treatment durations. The full description of this part is in another article.

The third part, which is reported in the present work, assessed the patients' QoL. Since GC-related side effects might influence the physical and mental disabilities of patients, we also included in this work some results of the second part.

We explored many potential physical and mental disabilities related to the disease and its treatment that might affect the patients' QoL. We thus developed close-ended questions (e.g., “At the disease onset, did you experience…?” or “Since the treatment start, did you …?”). Closed-ended questions were developed based upon the medical experience of the authors, who assess the abilities/disabilities of elderly patients daily, with the help of geriatricians. Moreover, some questions were retrieved from a literature review (11–16).

We also used the 12-item short form survey (SF-12) (QualityMetric Incorporated, License Number QM054800). The SF-12 survey explores physical, emotional and social health via assessment of physical activities, social activities, physical pain, general mental health, vitality and general health perception (17). In addition, the psychologic impact was assessed via the 30-item geriatric depression scale (GDS). The GDS added some items not explored in the SF-12 survey, especially regarding the consequences of an impaired mood. Moreover, this tool is especially appropriate to explore thymic states in elderly people.

In each patient, the SF-12 allowed us to calculate the physical score (PCS-12) and the mental score (MCS-12). A score ≥50 indicated no disability; 40-49: mild disability; 30–39: moderate disability; and <30: severe disability. We pooled together patients without and with mild disability on one side and those with moderate and severe disability on the other. Regarding the GDS, a score of 0–9 was normal, 10–19 suggested slight depression, and a score >19 was indicative of moderate to severe depression.

Based on the responses obtained in the second part of the questionnaire, we analyzed the specific impact of GC-related adverse events (AEs) on declared physical and mental disabilities.

Finally, we also asked patients to specify whether their physical autonomy, assessed via the ability to perform their usual daily activities, including walking, leaving the home, or climbing stairs, was affected since the disease diagnosis and its related treatment.

Data about baseline clinical manifestations and therapeutic management were retrieved via our centralized database.

### Statistical Analysis

Categorical variables are expressed as numbers (%), and quantitative variables are expressed as medians [range]. To compare the two groups, categorical variables were analyzed using the Pearson or Fisher Chi-square test as appropriate, and quantitative variables were analyzed using Wilcoxon's rank-sum test.

Logistic regression was used to determine which factors were the most associated with moderate-to-severe physical or mental disability. Odds ratios (ORs) and 95% confidence intervals (CIs) were computed for each factor in the univariate analysis and in the multivariate model with a backward stepwise approach using variables that reached *p* <0.2 in univariate analyses.

Spearman correlation coefficients were calculated to assess the correlation between GDS and PCS-12 and between GDS and MCS-12.

The statistical analyses were computed using JMP 9.0.1 (SAS Institute Inc., Cary, NC, USA). A p ≤ 0.05 defined statistical significance.

## Results

Among the 100 GCA patients solicited, 90 agreed to participate and sent back the completed questionnaire. The 10 patients who were not included were dead (*n* = 3), expressed a refusal to participate (*n* = 1) or did not send back the questionnaire (*n* = 6).

The 90 study participants were diagnosed with GCA from 2016 to early 2020, including 20 in 2016, 16 in 2017, 23 in 2018, 24 in 2019 and 7 in January 2020.

The median age of these 90 patients, among whom 71% were women, was 75 [60–94] years. The median follow-up since diagnosis was 20 [3–48] months, and 52 (58%) patients still received GC when completing the questionnaire. At the time of questionnaire completion, the overall GC median duration for the whole cohort, including patients who continued, was 17 [3–48] months. Twenty-nine (32%) patients received an immunosuppressant, methotrexate for 14 and tocilizumab for 15.

### Factors Associated With Moderate-to-Severe Physical Disability

**Figure 1:**
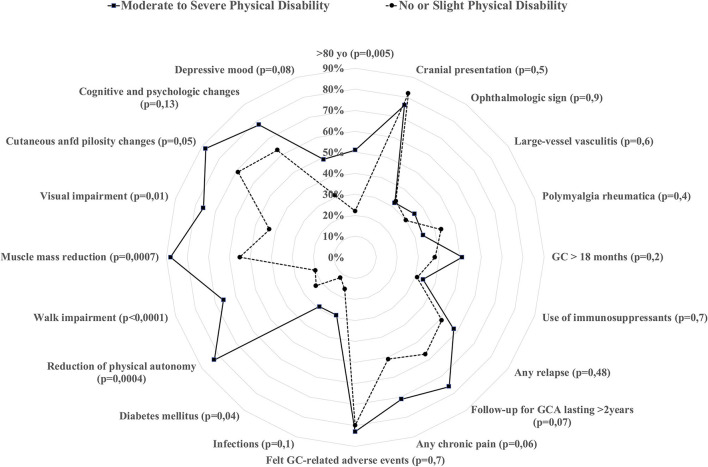
Comparison of different characteristics at GCA baseline and during follow-up according to whether the patients stated they suffered from moderate-to-severe physical disability (calculated through the SF-12 survey).

According to the SF-12, the median physical score was 41 [21–57]. Twenty-two (24%) patients had a score >50, i.e., did not report any physical disability; 27 (30%) reported a score between 40 and 49, i.e., expressed a mild physical disability; 28 (31%) reported a score between 30 and 39, i.e., a moderate physical disability; and 13 (14%) reported a score <30, indicative of a severe physical disability. Altogether, 49 (54%) patients expressed no or slight physical disability, whereas 41 others (46%) described moderate-to-severe physical disability. We compared these 2 groups in Table 1 and Figure 1.

**Table 1:** Comparison of GCA patients according to the felt severity of physical disability assessed by the SF-12 survey.

	**None-to-slight**	**Moderate-to-severe**	***P***
	**physical disability**	**physical disability**	
	**(*n* = 49)**	**(*n* = 41)**	
**Demographics**			
Age ≥80	11 (22)	21 (51)	0.005
Female	33 (67)	31 (76)	0.39
**Cardiovascular risk factors before GCA**			
≥2 cardiovascular risk factors	18 (37)	13 (32)	0.61
Coronaropathy	0	6 (15)	0.006
Any stroke before GCA	0	1 (2)	0.27
**GCA characteristics at diagnosis**			
Large-vessel vasculitis	14/47 (30)	14/40 (35)	0.60
Any cranial sign	40 (82)	31 (76)	0.49
Ophthalmologic sign	16 (33)	13 (32)	0.92
Uni- or bi-lateral blindness	4 (18)	4 (31)	0.39
Polymyalgia rheumatica	21 (43)	14 (34)	0.40
**GCA treatments and course**			
GC discontinuation at last follow-up	17 (35)	21 (51)	0.11
GC duration in all patients	17 [6–48]	19 [6–44]	0.24
GC duration of >18 months	19 (39)	21 (51)	0.24
Use of immune-suppressants	15 (31)	14 (34)	0.72
Any disease relapse	25 (51)	24 (58)	0.48
Total follow-up	17 [6–48]	21 [6–50]	0.11
Follow-up for GCA lasting >2 years	28 (57)	31 (76)	0.07
**Felt adverse events attributable to GC**	39 (80)	34 (83)	0.69
**Cardiovascular changes**	15 (31)	12 (29)	0.89
**Any metabolic complications**	23 (47)	21 (51)	0.69
Diabetes mellitus	6 (12)	12 (29)	0.04
Weight gain	20 (41)	16 (39)	0.86
**Muscle mass and strength reduction**	27 (55)	36 (88)	0.0007
**Cognitive and psychologic changes**	44 (90)	37 (90)	0.13
Memory loss	15 (30)	21 (51)	0.047
Depressive mood	15 (31)	20 (49)	0.08
Exalted mood	16 (33)	10 (24)	0.39
Insomnia	36 (73)	29 (70)	0.77
Irritability	25 (51)	17 (41)	0.37
**Osteoporotic fractures**	3 (6)	5 (12)	0.31
**Cutaneous and hairiness changes**	30 (61)	33 (80)	0.047
**Any infections requiring treatment**	9 (18)	14 (34)	0.09
**Any visual change**	15 (31)	23 (56)	0.01
Cataract	14 (29)	21 (51)	0.03
**Persisting articular pain**	24 (49)	30 (73)	0.02
**Reduction of physical autonomy**	23 (47)	34 (83)	0.0004
Need some help in daily activities	4 (8)	13 (32)	0.005
Mechanical fall	10 (20)	9 (22)	0.86
Walking difficulties	10 (20)	27 (66)	<0.0001

*Values are numbers (%) or medians [range]*.

*GCA, giant-cell arteritis; GC, glucocorticoids*.

At baseline, patients with moderate-to-severe physical disability more frequently were >80 years of age (51 vs. 22%, *p* = 0.005) and had coronaropathies (15% vs. none in the other group, *p* = 0.006). Although the rate of GC-related AEs was not different between the two groups, patients with moderate-to-severe physical disability developed more diabetes (29 vs. 12%, *p* = 0.04), more muscle and strength reduction (88 vs. 55%, *p* = 0.0007), and more visual changes (56 vs. 31%, *p* = 0.01). Patients with moderate-to-severe physical disability also reported reduced autonomy (83 vs. 47%, *p* = 0.0004), especially walking impairment (66 vs. 20%, *p* < 0.0001).

In Table 2, we identified through logistic regression the factors most associated with moderate-to-severe physical disability. Walk difficulties (*OR* = 8.42 [95% CI, 2.98–26.82], *p* < 0.0001), muscle mass and strength reduction (OR = 4.38 [1.3–16.31], *p* = 0.01) and age >80 years (OR = 4.21 [1.44–13.61], *p* = 0.008) were the 3 factors with the most negative impact on physical disability.

**Table 2:** Factors associated with moderate-to-severe physical disability in univariate and multivariate models.

	**Univariate OR**,	***P***	**Multivariate OR**,	***p***
	**95% CI**		**95% CI**	
Age >80	3.62 [1.49–9.26]	0.004	4.21 [1.44–13.61]	0.008
GC discontinuation	1.97 [0.85–4.68]	0.11		
GC duration >12 months	1.88 [0.78–4.72]	0.16		
GCA >2 years	2.32 [0.95–5.95]	0.06		
Diabetes mellitus	2.97 [1.03–9.36]	0.04		
Cutaneous and hairiness changes	2.61 [1.02–7.15]	0.04		
Muscle mass and strength reduction	5.87 [2.1–19.33]	0.0005	4.38 [1.37–16.31]	0.01
Persisting articular pain	2.32 [0.98–5.70]	0.06		
Memory loss	2.38 [1.01–5.73]	0.05		
Depressive mood	2.15 [0.92–5.19]	0.08		
Any infections requiring treatment	2.30 [0.88–6.26]	0.09		
Any visual change	2.90 [1.23–7.02]	0.01		
Walking difficulties	7.52 [3–20.23]	<0.0001	8.42 [2.98–26.82]	<0.0001

*Odds ratios (ORs) and 95% confidence intervals (CIs)*.

*GCA, giant cell arteritis; GC, glucocorticoids*.

### Factors Associated With Moderate-to-Severe Mental Disability

**Figure 2:**
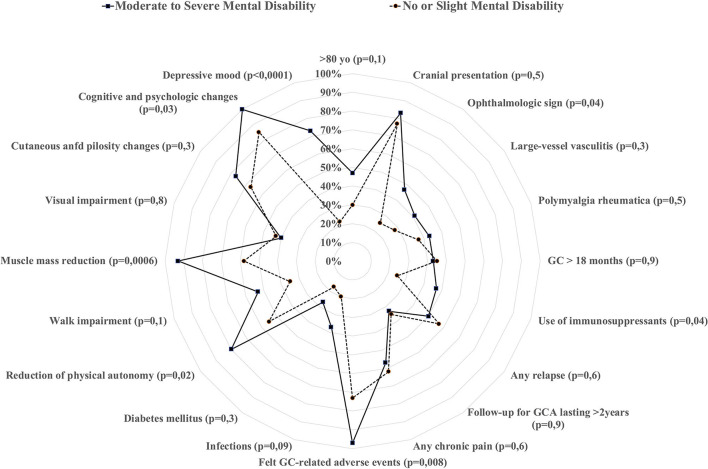
Comparison of different characteristics at GCA baseline and during follow-up according to whether the patients stated they suffered from moderate-to-severe mental disability (calculated through the SF-12 survey).

According to the SF-12, the median mental score was 46 [22–62]. Thirty-tree (37%) patients had a score ≥50, i.e., did not report any mental disability; 27 (30%) reported a score between 40 and 49, i.e., mild mental disability; 20 (22%) reported a score between 30 and 39, i.e., moderate mental disability; and 10 (11%) reported a score <30, indicative of a severe mental disability. Altogether, 60 (67%) had no or slight mental disability, and 30 (33%) described moderate-to-severe mental disability. We compared these 2 groups in Table 3 and Figure 2.

**Table 3:** Comparison of GCA patients according to the felt severity of mental disability as assessed by the SF-12 survey.

	**None-to-slight**	**Moderate-to-severe**	***p***
	**mental disability**	**mental disability**	
	**(*n* = 60)**	**(*n* = 30)**	
**Demographics**			
Age >80	18 (30)	14 (47)	0.12
Female	42 (70)	22 (73)	0.74
**Cardiovascular risk factors before GCA**			
>2 cardiovascular risk factors	21 (35)	10 (33)	0.88
Coronaropathy	3 (5)	3 (10)	0.37
Any stroke before GCA	0	1 (3)	0.16
**GCA characteristics at diagnosis**			
Large-vessel vasculitis	17 (28)	11 (41)	0.25
Any cranial sign	46 (77)	25 (83)	0.47
Ophthalmologic sign	15 (25)	14 (47)	0.04
Uni- or bi-lateral blindness	3 (13)	5 (42)	0.06
Polymyalgia rheumatica	22 (37)	13 (43)	0.54
**GCA treatments and course**			
GC discontinuation at last follow-up	24 (40)	14 (47)	0.55
GC duration in all patients	17 [6–48]	18 [6–44]	0.81
Total follow-up	19 [6–50]	24 [6–47]	0.33
GC duration of >18 months	27 (45)	13 (43)	0.88
Use of immunosuppressants	15 (25)	14 (47)	0.04
Any disease relapse	34 (57)	15 (50)	0.55
Follow-up for GCA lasting >2 years	21 (35)	10 (33)	0.88
**Felt adverse events attributable to GC**	44 (73)	29 (97)	0.008
**Cardiovascular changes**	13 (22)	14 (47)	0.01
**Any metabolic complications**	28 (47)	16 (53)	0.55
Diabetes mellitus	10 (17)	8 (27)	0.26
Weight gain	23 (38)	13 (43)	0.65
**Muscle mass and strength reduction**	35 (58)	28 (93)	0.0006
**Cognitive and psychologic changes**	51 (85)	30 (100)	0.03
Memory loss	22 (37)	14 (47)	0.36
Depressive mood	13 (22)	22 (73)	<0.0001
Exalted mood	13 (22)	13 (43)	0.03
Insomnia	41 (68)	24 (80)	0.24
Irritability	24 (40)	18 (60)	0.07
**Osteoporotic fractures**	4 (7)	4 (13)	0.29
**Cutaneous and hairiness changes**	40 (67)	23 (77)	0.33
**Any infections requiring treatment**	12 (20)	11 (37)	0.09
**Any visual change**	26 (43)	12 (40)	0.76
**Persisting articular pain**	37 (62)	17 (57)	0.65
**Reduction of physical autonomy**	33 (55)	24 (80)	0.02
Need some help in daily activities	10 (17)	7 (23)	0.45
Mechanical fall	11 (18)	8 (27)	0.36
Walk difficulties	21 (35)	16 (53)	0.1

*Values are numbers (%) or medians [range]*.

*GCA, giant-cell arteritis; GC, glucocorticoids*.

At baseline, patients who reported moderate-to-severe mental disability more frequently suffered from GCA-related ophthalmologic signs (47 vs. 25%, *p* = 0.04). They also reported more felt GC-related AEs (97 vs. 73%, *p* = 0.008), especially cardiovascular changes (47 vs. 22%, *p* = 0.01), muscle mass and strength reduction (93 vs. 58%, *p* = 0.0006), or depressive mood (73 vs. 13%, *p* < 0.0001). They also more frequently reported a reduction in their physical autonomy (80 vs. 55%, *p* = 0.02). Regarding therapeutic management, the GC durations (*p* = 0.81) were not different in either group, nor was the rate of relapse (*p* = 0.55). However, patients who reported moderate-to-severe mental disability more frequently received an immunosuppressant (47 vs. 25%, *p* = 0.04). Among the 29 patients who received an immunosuppressant, 7/14 (50%) who received methotrexate vs. 7/15 (47%) who received tocilizumab described moderate-to-severe mental disability (*p* = 1).

In Table 4, we identified via logistic regression the factors most associated with moderate-to-severe mental disability. Depressive mood (OR = 11.05 [95% CI, 3.78–37.11], *p* < 0.0001), felt GC-related AEs (OR = 10.54 [1.65–213.1], *p* = 0.01) and the use of an immunosuppressant (OR = 3.50 [1.14–11.87], *p* = 0.03) were the 3 factors with the most negative impact on mental disability.

**Table 4:** Factors associated with moderate-to-severe mental disability in univariate and multivariate models.

	**Univariate OR**,	***P***	**Multivariate OR**,	***p***
	**95% CI**		**95% CI**	
Age >80	2 [0.82–5.09]	0.12		
Ophthalmologic sign at diagnosis	2.63 [1.04-6.71]	0.04		
Use of immunosuppressants	2.53 [1.02–6.37]	0.04	3.50 [1.14–11.87]	0.03
Felt adverse events attributable to GC	10.5 [1.98–195.4]	0.003	10.54 [1.65–213.1]	0.01
Cardiovascular complications	3.16 [1.24–8.28]	0.02		
Reduction of physical autonomy	3.27 [1.23–9.88]	0.02		
Walking difficulties	2.12 [0.87–5.25]	0.1		
Muscle mass and strength reduction	10 [2.66–65.5]	0.0002		
Cognitive and psychologic changes	6 [1.85–27.09]	0.002		
Depressive mood	9.94 [3.74–28.96]	<0.0001	11.05 [3.78–37.11]	<0.0001
Exalted mood	2.76 [1.07–7.24]	0.04		
Irritability	2.25 [0.93–5.62]	0.07		
Any infections requiring treatment	2.32 [0.87–6.20]	0.09		

*Odds ratios (ORs) and 95% confidence intervals (CIs)*.

*GCA, giant cell arteritis; GC, glucocorticoids*.

### Psychologic Impact Assessed via the 15-Item Geriatric Depression Scale

Among the 90 patients, 16 (18%) did not have any sign of mood disorder, 72 (80%) had slight depression and 2 (2%) had moderate-to-severe depression. The Pearson correlation with the associated p-value was calculated between the GDS and the PCS-12 and the GDS and the MCS-12. There was a statistically significant negative correlation between GDS and the physical and/or mental disability scores (GDS and PCS-12: *r* = −0.33, *p* = 0.0013; GDS and MCS-12: *r* = −0.36, *p* = 0.0005).

## Discussion

The impact of the chronic course of GCA and its prolonged treatment on patients' QoL has been poorly analyzed. In the present study, we showed that approximately one-third to half of patients reported a physical and/or mental disability attributable to GCA and its treatment in the months or years following diagnosis. We observed that the described physical disabilities were not directly associated with GCA manifestations or with treatment management. Conversely, reductions in muscular mass and strength, walk impairment and visual deterioration were strongly associated with the severity of physical disability. However, even though these comorbidities are potentially linked or worsened by GC use, they should also be the consequence of natural aging, which is emphasized by the older age of patients with severe physical disability. Walking difficulties, and more extensively impairment of mobility, are reported in a few GCA studies and lead to a reduction of the physical autonomy and the ability to ensure daily activities such as self-care, dressing, washing, or shopping, which is concordant with our study (11, 12). Other studies have reported the negative impact of GCA and its treatment on some patients' ability to work, practice usual hobbies or leisure activities (12, 13). Altogether, these findings suggest paying particular attention to maintaining muscular autonomy and physical activities in the oldest patients, and encourage us to propose muscle reinforcement programs for these patients.

In accordance with others (12, 14, 15), our study showed that mental disability was worsened by GCA-related ophthalmologic impairment. Interestingly, patients also reported the negative mental impact of treatments, especially due to GC and immune-suppressants. In some of the studies where GCA patients were directly interviewed, they reported that GC increased their stress and anxiety, possibly leading to social isolation (12, 15). The mental assessment via the SF-12 survey and the GDS indicated that >80% of patients showed some signs of mood disorders. Other studies confirmed reduced self-esteem in GCA patients with a negative perception of their health and the feeling of not living a normal life (12, 13).

Many other factors, independent of GCA and its treatment, might be related to this thymic decline. However, this observation suggests the importance of thymic evaluation in GCA patients.

Based on our results, two main points should be highlighted. First, regardless of the disease status and its treatment, our patients showed an altered QoL, especially when aged >80. Although the exact role of GCA and its treatment cannot be precisely assessed in a global QoL evaluation, some targetable and measurable clinical and social parameters can be routinely checked during follow-up, such as physical autonomy or muscle mass maintenance.

Even though not directly assessed in this study, optimal management of GC to reduce AEs should remain a priority. Additionally, this study emphasizes the need for patient-reported outcome measures to evaluate the GC effect, which is in accordance with a recent study (16). Therefore, different international initiatives are planned to improve outcome measurement, especially through OMERACT programs (16–19).

The second main point regards the multidisciplinary approach required to correctly manage GCA patients. In addition to disease evaluation and treatment management, physicians should integrate the geriatric dimension of some GCA patients. Other actors, such as geriatricians, psychologists or psychiatrists, physiotherapists, in-home caregivers and therapeutic education professionals should be integrated into the care pathway of GCA patients.

Although our study is one of the few reporting patient outcomes through a self-evaluation in GCA, some points should be acknowledged and might reduce the validity of our observations. First, in the absence of a control group, the patients reported some symptoms that they attributed to the disease or its treatment, but no firm confirmation could be made. Although we observed an impaired QoL in many patients, we cannot conclude that their QoL was more impaired than other aged-matched healthy people. However, the first goal of this study was to provide a descriptive picture of the medical and social impacts of the disease and its treatment in the daily lives of GCA patients. Given the methodology used, each patient completed the questionnaire at different times of their disease and treatment, which can influence some results. However, we did not find any association between the disease or treatment durations and the disabilities. In addition to validated scales (SF-12, GDS), some of the questions addressed to patients were developed from our own experience and were not all replicated in other studies. The reduction of physical autonomy or the impact of muscle mass reduction can be linked to other important factors, such as aging, and may be independent of GCA and treatment. Second, some recall biases are likely. Given the old age of some of the patients and the possible cognitive-associated troubles, some symptoms should have been added or forgotten; however, the potential help of familial caregivers should have reduced this bias. The impact of treatment only focused on GC, but some patients also received immune-suppressants that can add some AEs, which were not assessed in this study. Immuno-suppressants probably have an impact since we showed that patients with a concomitant immunosuppressant had a more important mental disability, regardless of the type of immunosuppressant, i.e., methotrexate or tocilizumab.

To conclude, our study shows that GCA patients' QoL is frequently impaired by the disease or its treatment, regardless of the intrinsic favorable benefit of the latter. Important reported factors reflecting a severe disability, such as walking difficulties, muscle mass reduction, and glucocorticoid-related adverse events, were revealed by this study and are modifiable by medical and home care. Further studies, especially with a control group, are required to confirm our results and reinforce knowledge about disease-modifiable factors that affect patients' QoL.

## Data Availability Statement

The raw data supporting the conclusions of this article will be made available by the authors, without undue reservation.

## Ethics Statement

The studies involving human participants were reviewed and approved by CLERS-CAEN. The patients/participants provided their written informed consent to participate in this study.

## Author Contributions

HdB designed the study, analyzed the data, and wrote the manuscript. HdB, CB, AD, JB, NMS, GM, AN, AL, PC, SG, AS, SD, and AA collected the data and critically revised and edited the manuscript. All authors contributed to the article and approved the submitted version.

## Conflict of Interest

The authors declare that the research was conducted in the absence of any commercial or financial relationships that could be construed as a potential conflict of interest.

## Publisher's Note

All claims expressed in this article are solely those of the authors and do not necessarily represent those of their affiliated organizations, or those of the publisher, the editors and the reviewers. Any product that may be evaluated in this article, or claim that may be made by its manufacturer, is not guaranteed or endorsed by the publisher.
