# Supplementary material for: Impact of Giant Cell Arteritis and Its Treatment on the Patient's Quality of Life: A Single-Center Self-Assessment Study
Source: Front Med (Lausanne). 2021 Nov 10;8:777310. doi: 10.3389/fmed.2021.777310 (PMC8631395; doi:10.3389/fmed.2021.777310)
Supplement: Supplementary file 1 [file Data_Sheet_1.PDF]

PART II.

In this part of the questionnaire, we aim to analyze the impact of glucocorticoids on your health according to your own point of view.

TREATMENT

Regarding the corticosteroids use :

Before starting the treatment, did you have any concern regarding glucocorticoids ?

Not at all A little A lot

Did you feel better after glucocorticoids introduction ?

Not at all A little A lot

Did you experiment some adverse events you attributed to the glucocorticoids ?

Not at all A little A lot

Once glucocorticoids started, did you experiment one of these effects:

New or worsened hypertension Yes No

New or worsened diabetes Yes No

Weight gain Yes No

If yes, how much weight (compared to your usual weight) did you gain : …………….. Kgs

Loss of muscular strength Yes No

Fall(s) Yes No

New bone fracture Yes No

If yes, please detail:

Memory loss Yes No

Depressive mood Yes No

Exalted mood Yes No

Sleeping difficulties Yes No

Irritability	Yes	No
Cutaneous frailty	Yes	No
Cutaneous ecchymosis	Yes	No
Changes in pilosity	Yes	No
Infections	Yes	No

<u>If yes :</u>

Which infection(s) did you have: …………………………………………….

Were you hospitalized for the infection	Yes	No
Did you receive any antibiotics	Yes	No
Did you have a zona	Yes	No

Vision loss	Yes	No

<u>If yes :</u>

New or worsened cataract	Yes	No
New or worsened glaucoma	Yes	No

Did you modify your eating diet?	Yes	No

<u>Please, list the other effects you observed once glucocorticoids were started</u>

……………………………………………………………………………………………………

……………………………………………………………………………………………………

……………………………………………………………………………………………………

……………………………………………………………………………………………………

For you, what was the main side effect of glucocorticoids ?

…………………………………………………………….

PART III.

In this part of the questionnaire, we aim to analyze the impact of the disease and its treatment on your daily life. Please consider your response keeping in mind that we wish to analyze your daily life since your GCA was diagnosed.

Since the GCA diagnosis and the treatment introduction :

Did you lose any physical autonomy (e.g. performing your usual daily activities, including walking)?

Yes No

Did you leave your home to go outdoors ? Yes No

Did you experience difficulties walking ? Yes No

Did you request assistance ?

To groom yourself Yes No

To prepare meals Yes No

To dress Yes No

To do your shopping Yes No

Which activities are impacted by the disease and its treatment?

…………………………………………

…………………………………………

…………………………………………

…………………………………………

…………………………………………

Did you experience any memory loss? Yes No

If yes,

Did somebody in your social circle notice? Yes No

Have you consulted a specialized medical doctor? Yes No

Did you feel depressed ? Yes No

Currently (when you fill out this questionnaire) :

Your disease is : inactive moderately active active

Do you (still) have headaches ? Yes No

Do you (still) have articular pain that wakes you at night? Yes No

Quality of life questionnaire

1. **In general, would you say your health is**:

☐1 Excellent ☐2 Very good ☐3 Good ☐4 Fair ☐5 Poor

The following questions are about activities you might do during a typical day. Does your health now limit you in these activities? If so, how much?

	**YES, limited a lot**	**YES, limited a little**	**NO, not limited at all**
2. **Moderate activities** such as moving a table, pushing a vacuum cleaner, bowling, or playing golf.	☐1	☐2	☐3
3. Climbing **several** flights of stairs.	☐1	☐2	☐3

During the past 4 weeks, have you had any of the following problems with your work or other regular daily activities as a result of your physical health?

	**YES**	**NO**
4. **Accomplished less** than you would like.	☐1	☐2
5. Were limited in the **kind** of work or other activities.	☐1	☐2

During the past 4 weeks, have you had any of the following problems with your work or other regular daily activities as a result of any emotional problems (such as feeling depressed or anxious)?

	**YES**	**NO**
6. **Accomplished less** than you would like.	☐1	☐2
7. Did work or activities **less carefully than usual**.	☐1	☐2

8. **During the past 4 weeks, how much did pain interfere with your normal work (including work outside the home and housework)?**

☐1 Not at all ☐2 A little bit ☐3 Moderately ☐4 Quite a bit ☐5 Extremely

These questions are about how you have been feeling during the past 4 weeks.
For each question, please give the one answer that comes closest to the way you have been feeling.

How much of the time during the past 4 weeks…

	All of the time	Most of the time	A good bit of the time	Some of the time	A little of the time	None of the time
9. Have you felt calm & peaceful?	☐1	☐2	☐3	☐4	☐5	☐6
10. Did you have a lot of energy?	☐1	☐2	☐3	☐4	☐5	☐6
11. Have you felt down-hearted and blue?	☐1	☐2	☐3	☐4	☐5	☐6

12. **During the past 4 weeks, how much of the time has your physical health or emotional problems interfered with your social activities (like visiting friends, relatives, etc.)?**

☐1 All of the time ☐2 Most of the time ☐3 Some of the time ☐4 A little of the time ☐5 None of the time

Questionnaire about your mood

1.	Are you basically satisfied with your life?	Yes	**No (1)**
2.	Have you dropped many of your activities and interests?	**Yes (1)**	No
3.	Do you feel that your life is empty?	**Yes (1)**	No
4.	Do you often get bored?	**Yes (1)**	No
5.	Are you hopeful about the future?	Yes	**No (1)**
6.	Are you bothered by thoughts you can't get out of your head?	**Yes (1)**	No
7.	Are you in good spirits most of the time?	Yes	**No (1)**
8.	Are you afraid that something bad is going to happen to you?	**Yes (1)**	No
9.	Do you feel happy most of the time?	Yes	**No (1)**
10.	Do you often feel helpless?	**Yes (1)**	No
11.	Do you often get restless and fidgety?	**Yes (1)**	No
12.	Do you prefer to stay at home rather than go out and do things?	**Yes (1)**	No
13.	Do you frequently worry about the future?	**Yes (1)**	No
14.	Do you feel you have more problems with memory than most?	**Yes (1)**	No
15.	Do you think it is wonderful to be alive now?	Yes	**No (1)**
16.	Do you feel downhearted and blue?	**Yes (1)**	No
17.	Do you feel pretty worthless the way you are now?	**Yes (1)**	No
18.	Do you worry a lot about the past?	**Yes (1)**	No
19.	Do you find life very exciting?	Yes	**No (1)**
20.	Is it hard for you to get started on new projects?	**Yes (1)**	No
21.	Do you feel full of energy?	Yes	**No (1)**
22.	Do you feel that your situation is hopeless?	**Yes (1)**	No
23.	Do you think that most people are better off than you are?	**Yes (1)**	No
24.	Do you frequently get upset over little things?	**Yes (1)**	No
25.	Do you frequently feel like crying?	**Yes (1)**	No
26.	Do you have trouble concentrating?	**Yes (1)**	No
27.	Do you enjoy getting up in the morning?	Yes	**No (1)**
28.	Do you prefer to avoid social occasions?	**Yes (1)**	No
29.	Is it easy for you to make decisions?	Yes	**No (1)**
30.	Is your mind as clear as it used to be?	Yes	**No (1)**
